# Supplementary material for: Causes and consequences of sex-chromosome turnovers in Diptera
Source: Evol Lett. 2026 Mar 12;10(3):253–68. doi: 10.1093/evlett/qrag003 (PMC13231274; doi:10.1093/evlett/qrag003)
Supplement: qrag003_Supplemental_Files [file qrag003_supplemental_files.zip › Supplementary_Datasets.zip!/Supplementary_Datasets/Dataset_2_Graphical_representation_outgroups/Culicidae_Anopheles_marshallii_chromosomes.pdf]

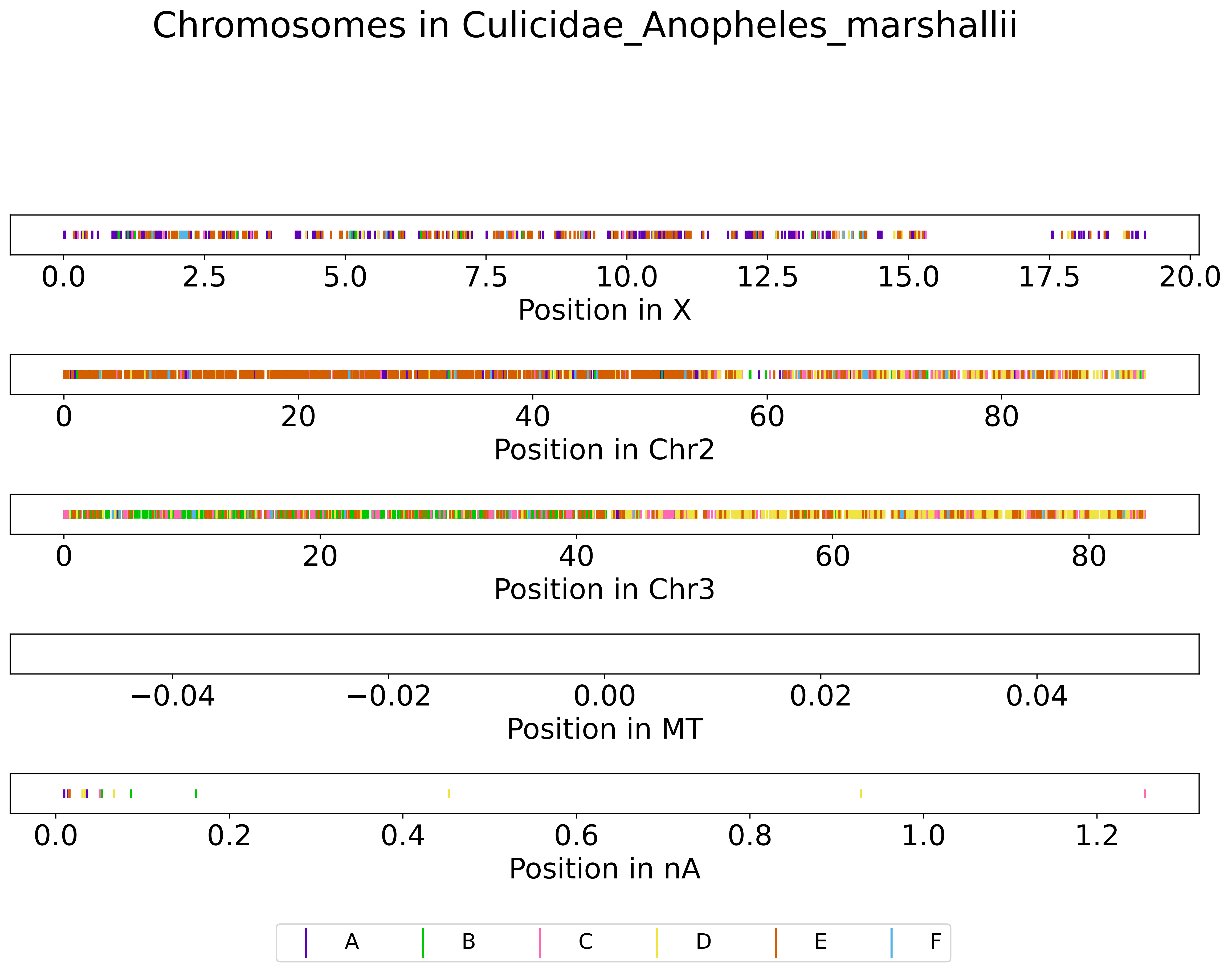
Chromosomes in Culicidae_Anopheles_marshallii
0.0
2.5
5.0
7.5
10.0
12.5
15.0
17.5
20.0
Position in X
0
20
40
60
80
Position in Chr2
0
20
40
60
80
Position in Chr3
−0.04
−0.02
0.00
0.02
0.04
Position in MT
0.0
0.2
0.4
0.6
0.8
1.0
1.2
Position in nA
A
B
C
D
E
F
